# Analysis of a Film Forming Process through Coupled Image Correlation and Infrared Thermography

**DOI:** 10.3390/polym14061231

**Published:** 2022-03-18

**Authors:** Moritz Neubauer, Martin Dannemann, Niklas Herzer, Benjamin Schwarz, Niels Modler

**Affiliations:** 1 Institute of Lightweight Engineering and Polymer Technology (ILK), Technische Universität Dresden, Holbeinstraße 3, 01307 Dresden, Germany; martin.dannemann@fh-zwickau.de (M.D.); niklas.herzer@mailbox.tu-dresden.de (N.H.); niels.modler@tu-dresden.de (N.M.); 2 GOM GmbH, Schmitzstraße 2, 38122 Braunschweig, Germany; b.schwarz@gom.com

**Keywords:** digital image correlation, infrared thermography, strain localization, thermomechanical behavior, film, thermoplastic, bending, thermoforming

## Abstract

The aim of the present investigation was to determine the dependence of the material and process parameters of the bending process of thermoplastic films. In this context, parameter combinations leading to high resulting forming ratios were identified. To measure the relevant parameters within the hot bending process, a coupled evaluation of infrared thermography (IRT) and deformation measurement using digital image correlation (DIC) was performed. The coupled measurement enables the identification of the actual mechanically stressed bending area of the film as a result of the bending process. This allows for the specification of the local forming temperatures required for the desired forming ratios. Furthermore, the mechanical and thermal strain along the defined measuring sections and their deviation in individual tests as well as the effect of thermal strain on process control on a larger scale were determined. Based on the results, a process window was defined for the film materials investigated, which will serve as a starting point for future efforts to develop a continuous manufacturing process.

## 1. Introduction

### Motivation

In current studies, novel cellular hybrid composite structures have been investigated for the application in sound-absorbing liners for aerospace applications [1,2]. Besides the Helmholtz resonator effect, the described structures utilize flexible wall areas with high intrinsic material damping to enhance acoustic performance [3]. In this context, the material group of thermoplastics and thermoplastic elastomers seems to be well suited for the flexible walls due to their damping behavior, which could lead to a higher overall damping performance of the resonator. In order to increase the stability of the structure, rigid walls consisting of fiber-reinforced plastics are integrated in each cell, serving as a support structure.

For the production of such cellular structures, various manufacturing processes exist, where the relevant technologies are described in the following. The choice of process depends, among other things, on the materials used, the geometric properties, and the desired quantity. For instance, roll welding processes are used in the manufacture of metallic honeycomb structures [4,5]. In addition, there are a number of additive manufacturing processes that enable the production of cellular core structures for metallic and plastic materials [6,7,8]. In this context, Zaharia et al. [9] analyzed the cellular core structures fabricated by material extrusion-based additive manufacturing using biodegradable materials. Furthermore, past investigations have shown the potential to transfer cellular structures into a continuous production [10,11]. However, these manufacturing processes lack the integration of a support structure, which is required for applications such as an acoustic liner.

**Figure 1:**
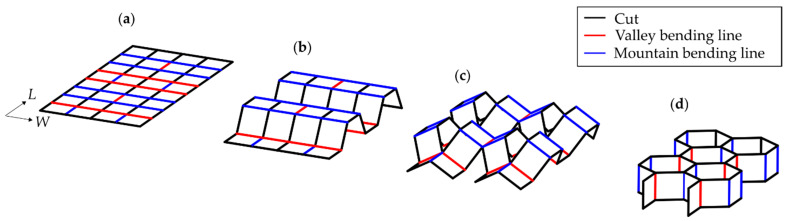
(**a**) Flat film sheet with designated bending lines, (**b**) applying bends in the W-direction, (**c**) applying cuts and bends in the L-direction, and (**d**) final cellular structure.

In the context of a continuous process, the molding procedure, besides the integration of the support structure, is a critical step due to the complex geometry of the cellular structures. For the realization of complex geometries, cost-effective thermoforming is a predestined process for thin-walled components made of polymers, which are widely used in various industries such as mobility, packaging, and medicine [12,13,14,15,16]. A method for the continuous fabrication of cellular structures was described by Fan et al. [17], which allows for a more cost-effective production of honeycomb structures for structural applications due to the continuous fabrication technology. The generic term of thermoforming usually describes the heating process of a large portion of the polymer sheet and the subsequent differential stretching process, resulting in a thermoformed part with usually non uniform wall thicknesses [18]. A process that has received little attention to date is the continuous hot bending of films, where local heating and forming takes place along the bending line as opposed to full-surface heating and forming. In addition, the material thickness of the molded part produced remains almost constant in relation to the initial thickness of the semi-finished product [19]. Recently, the design and fabrication of cellular structures and the corresponding fabrication devices used for the production of single-curvature folded cores produced by bend-like processes have been described, showing the great potential of these folding processes [20,21,22]. A transfer of this approach into a continuous process chain for folding thermoplastic honeycomb core structures has been described by Kucher et al. [23]. Figure 1 shows an example of the manufacturing steps for fabricating the established cellular honeycomb structure by the targeted bending technique.

This process could hold the potential to integrate the above-mentioned support elements into the structure and represents the targeted process for the presented bending tests. In this context, the underlying mechanism of the hot bending process of thermoplastic films and the corresponding crucial process parameters need to be investigated.

With respect to the measurement and analysis of deformations and processing temperature, the non-contact measurement techniques of digital image correlation (DIC) and infrared thermography (IRT) show high potential [24,25,26,27]. IRT is a widely used method for non-contact, full-field temperature measurement with minimal instrumentation that is required for the presented evaluation of the temperature distribution on the film surface during the bending process [28]. The DIC technique is accurate in measuring small to medium–large strains while allowing for the determination of displacement components in all three spatial directions simultaneously [29,30,31]. The fact that DIC has a lower susceptibility to vibration than, for example, electronic speckle pattern interferometry, is beneficial for the targeted investigation. The coupling of both of these methods can be applied to a large spectrum of actual problems by enabling the acquisition of temperature and displacements at each spatial point at each acquired time step [27]. A large proportion of the available research has focused on thermomechanical response measurements associated with heat generation during the elastic and plastic deformation of strain probes differing in the analyzed material and strain rates—levels and scale [26,27,32,33,34,35]. Besides the application in material characterization, the DIC method has been applied in order to analyze forming processes such as the thermoforming process of thermoplastic sheets and films in order to identify suitable process parameter ranges to ensure high product quality [36,37,38]. However, there have only been a few research items focusing on the combination of DIC and IRT for the assessment of molding processes [39,40].

In the scope of this paper, a method for analyzing the hot bending process of thermoplastic film materials is presented. In order to determine the relevant forming ratio, bending area, and forming temperature parameters, the coupled DIC and IRT measurement method was applied. Based on the experimental results, a favorable range of the relevant process parameter is presented to serve as a basis for future studies on the molding of thermoplastic films in a continuous process.

## 2. Materials and Methods

In the course of the presented study, two film materials were investigated. These were thermoplastic polyurethane (TPU), which refers to the group of materials known as thermoplastic elastomers (TPE), and polypropylene (PP), referring to a group of thermoplastics (see Table 1). The polyether-based TPU (ELASTOLLAN 1170 A, BASF Polyurethanes GmbH, Lemförde, Germany,) was selected since in previous studies on a novel acoustic liner, the application of TPU film resulted in improved sound absorption compared to conventional liner structures due to its damping properties [2,41]. The PP material (NOLAX A21.4500, Nolax AG, Sempach, Switzerland) was chosen as a reference material due to its versatility and ease of processing as well as its potential application in acoustic liners.

**Figure 2:**
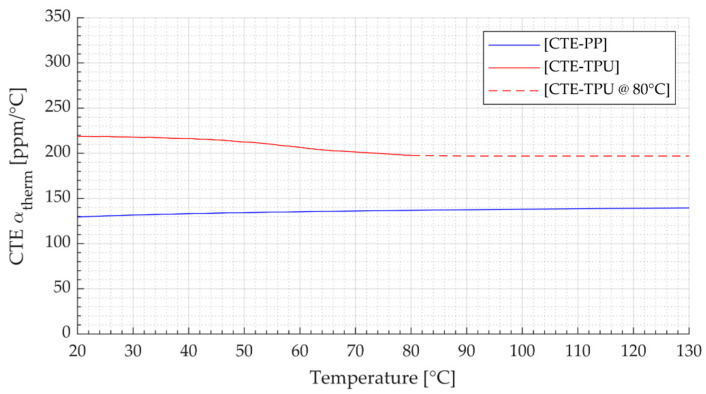
CTE for TPU according to manufacturer’s specifications within the range 20 °C to 80 °C (CTE-TPU, red line), retained value of CTE of TPU at 80 °C for the remaining temperature range (CTE-TPU @ 80 °C, red dashed line), and experimentally determined CTE of PP (CTE-PP, blue line).

To enable the detection of surface deformation with the 3D DIC system, a thin speckle pattern was applied on the film specimens with developer spray, reducing its effect on the expansion of the specimens. For the prepared specimens, the emissivity was determined in a comparative measurement experiment with a thermocouple over the temperature range from 20 °C to 130 °C. The measurement with the thermocouple and the IR camera yielded analog temperature values compared to the data acquired by IRT with a maximum deviation of ±1 K, which was within the measurement uncertainty of the IR camera. In this context, an average emission coefficient of 1.0 was determined and used for the following analyses. The temperature-dependent coefficient of thermal expansion (CTE) $\alpha_{therm}$ for TPU within the temperature range of 20 °C to 80 °C was drawn from the manufacturer’s specifications [42]. For the remaining range from 80 °C to 130 °C, the CTE value of the TPU material at 80 °C was retained. For PP, the CTE was determined in a test with coupled DIC and IRT (see Figure 2).

**Figure 3:**
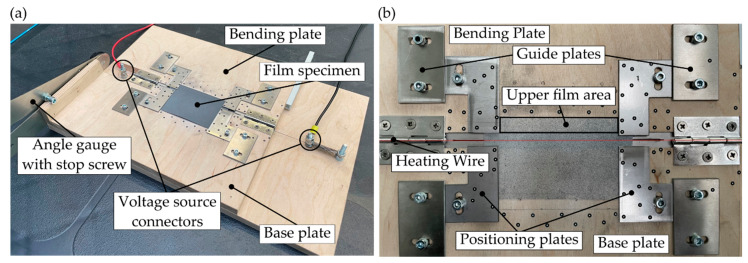
(**a**) Bending device with clamped film specimen and power supply and (**b**) top view of the components of the plate that enabled adjustments within the bending process.

In the remainder of the study, the films are referred to by their material and thickness, where TPU-0.3 refers to the thermoplastic polyurethane film with a thickness of 0.3 mm. The rectangular geometry of the investigated film specimens is shown in Figure 3b using PP-0.1 as an example.

### 2.1. Bending Device

The experimental apparatus designed for the hot bending tests was based on the principle of a commercially available heating line bending machine. By means of an integrated heating wire and subsequent manual forming through bending of the semi-finished product, simple forming processes can be carried out with thermoplastic sheets or films (see Figure 3).

At the beginning of the bending process, the adjustable bending plate remains horizontal so that the surfaces of the base and the bending plate are aligned in one plane. After the film has been positioned, it is fixed by tightening the screws in the positioning plates. The heating wire is located between the base plate and the bending plate, so that the film can be heated on a linear surface on one side. One end of the heating wire is connected to a pretensioned spring to compensate for temperature-induced length variations. The angle gauge with an integrated stop screw is used for a first adjustment of the maximum angle between the two plates. The exact angle is determined with the aid of the optical measuring system in the subsequent test evaluation. The bending radius can be adjusted by changing the distance between the opposing positioning plates before each bending cycle. The guide plates ensure that the positioning plates maintain a perpendicular position to the heating wire.

### 2.2. Experimental Setup and Measuring Equipment

**Figure 4:**
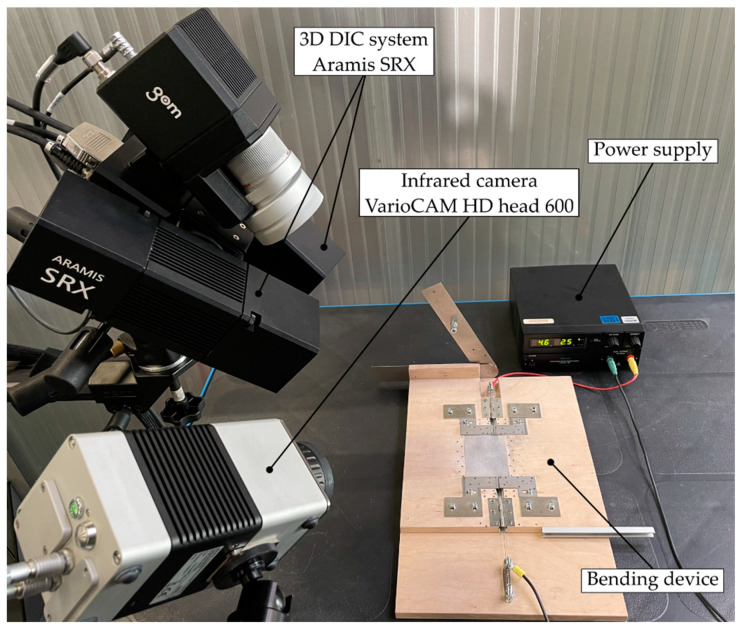
Experimental setup for deformation quantification and synchronous acquisition of the temperature field during the bending process.

For the analysis of the bending procedure, the film specimens were synchronously monitored using an infrared (IR) camera and a 3D DIC system. The IR camera (VarioCAM HD head 600, INFRATEC GmbH, Dresden Germany) was used to capture the local time resolved temperature evolution on the upper surface of the film facing away from the heating wire with a resolution of 0.025 °C and a measurement accuracy of ±1 K. The 640 × 480 pixel sensor supports the acquisition of freely configurable partial images from 60 Hz, the full image to 120 Hz, and 240 Hz in the cross-sectional image with reduced resolution. The temperature data were acquired over the entire measurement period. The 3D DIC system (ARAMIS SRX, GOM GmbH, Braunschweig, Germany) with a resolution of 4096 × 3068 pixels was used to measure the local strains on the upper surface of the film in a calibrated measurement volume with the dimensions of 180 mm × 155 mm × 65 mm at 75 fps. The sensor was capable of framerates up to 2000 fps with reduced image height. The field of view of the IR camera and the measurement volume of the ARAMIS SRX were aligned to the same measuring scope and were coupled via a trigger connection for synchronous image acquisition. The experimental setup included the bending device, the power supply, the IR camera, and the 3D DIC system (see Figure 4).

### 2.3. Motivation for a Coupled DIC-IRT Measurement

In order to carry out a profound investigation of the forming process of the thermoplastic films by means of hot bending, it is necessary to establish a coupling between the 3D DIC system and the IR camera. Thereby, one goal is to locate the area that is subject to mechanical deformation during bending. Furthermore, the location-dependent determination of the temperature at the time of bending needs to be enabled in order to identify a temperature range that leads to a high resulting forming ratio. The coupled measurement thus provides precise information on the location of the bending area and enables the location-dependent temperatures to be evaluated. This forms the basis of a simultaneous all-in-one measurement of various process parameters.

### 2.4. Test Procedure

To ensure synchronous image acquisition, the IR camera was triggered by a 5 V trigger-out signal of the ARAMIS SRX. Before the start of each measurement procedure, a reference image with the corresponding thermography data was acquired. The measurement started with a measuring frequency of 5 Hz while the power supply was turned on and the film was heated for the defined heating duration $t_{heat}$. At the end of the heating period, the bending of the film was performed by manually lifting the adjustable bending plate to the defined bending angle given by the attached stop screw of the angle gauge (see Figure 3). The goal was to achieve a uniform bending speed for the targeted angle with a uniform setting time of 3 s. As soon as the film was set to the final bending angle, the power supply was switched off and the measuring frequency of both measurement systems was reduced to 0.2 Hz until the end of the measurement to limit the amount of data. At the same time, the maximum value of the temperature films surface was monitored. After the positioning plates were removed and the maximum surface temperature dropped below 35 °C, the bending plate was placed horizontally. From this point, the measurement continued at the same measuring frequency of 0.2 Hz for five minutes until the end of the procedure.

### 2.5. Determination of the Bending Angles

**Figure 5:**
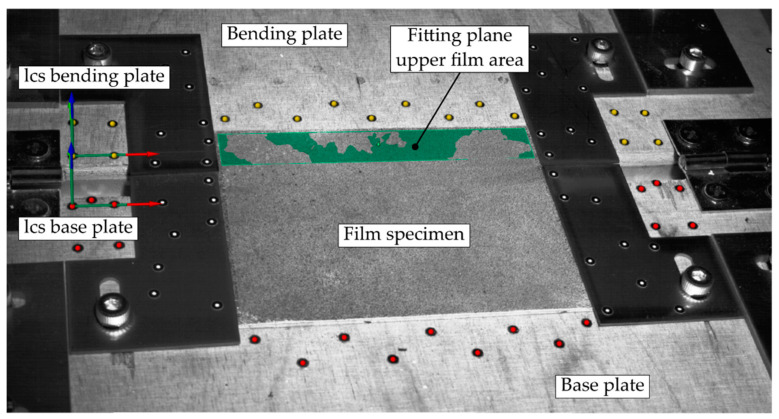
Measurement recording of the ARAMIS SRX before the start of the measurement procedure with local coordinate systems (lcs) and markers to track the position of the components during the bending process as well as the created fitting plane of the upper film area to determine the forming ratio.

In order to calculate the forming ratio of the film after bending, the surfaces of the involved appliances were marked with a stochastic speckle pattern, enabling the definition of corresponding planes and deformation tracking in the time-based component analysis software GOM ARAMIS Professional (see Figure 5).

**Figure 6:**
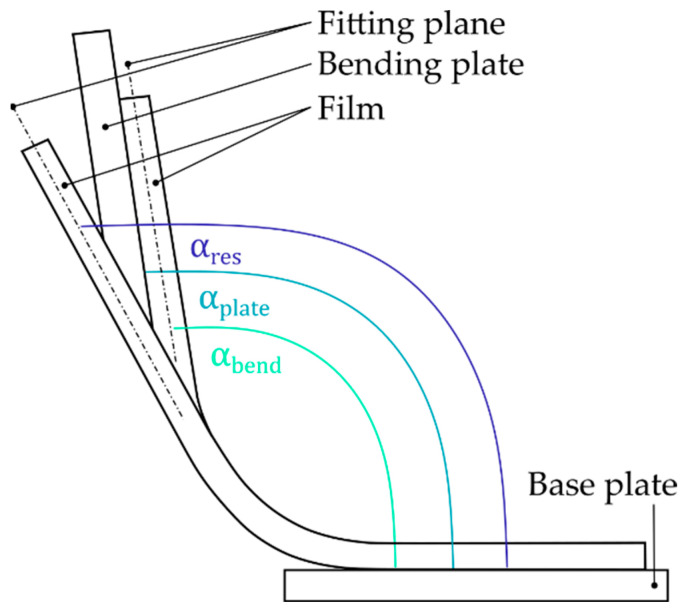
Illustration of the relevant angles for determining the forming ratio $\theta_{res}$ of the film.

In order to calculate the bending angle, the corresponding planes of the bending plate (yellow markers) and the base plate (red markers) were generated. To compensate for the waviness of the film material and to reduce measuring uncertainties, a fitting plane (green) was calculated for the film area, which was bent upward in the later course of the bending process. It is therefore defined as the upper film area and includes the part of the film that rests on the bending plate. With the defined planes, the relevant angles can be determined in order to calculate the resulting forming ratio (see Figure 6).

In this context, the plate angle ($\alpha_{plate}$) refers to the angle that spans between the base plate and the bending plate in its final position within the bending process. Consequently, the bending angle ($\alpha_{bend}$) refers to the angle between the base plate and the film (fitting plane) at the same time the plate angle is determined. The resulting film angle ($\alpha_{res}$) corresponds to the angle between the base plate and the final position of the film (fitting plane) after the positioning plates (see Figure 3b) had been removed, the bending plate had been laid down, and the film had cooled off. The forming ratio $\theta_{res}=\alpha_{res}/\alpha_{bend}$ was defined as the ratio of the resulting and the bending angle.

### 2.6. Determination of the Bending Area

In the course of the bending process, a mechanical deformation of the film specimens occurs. The area where this mechanical deformation occurs is called the bending area and comprises the section of the film where the deformation exceeds a defined limit. In order to be able to separate the area in which mechanical deformation takes place, the total elongation of the film must first be determined. Then, the temperature-induced thermal strain is calculated. If the thermal strain is subtracted from the total strain, the mechanical deformation of the film in the corresponding area is obtained.

In this context, it is important to mention that from the beginning of the test until the initiation of the bending procedure, a pre-deformation of the film occurs. This deformation is due to the experimental setup and is related to the temperature-induced change in the structural properties of the film. With increasing temperature, the films stiffness decreases, leading to an elongation of the film in the direction of the gravitational force. Therefore, the measured strain between the reference-measuring step before lifting the bending plate and the measuring step after reaching the targeted bending angle were considered to determine the bending area.

**Figure 7:**
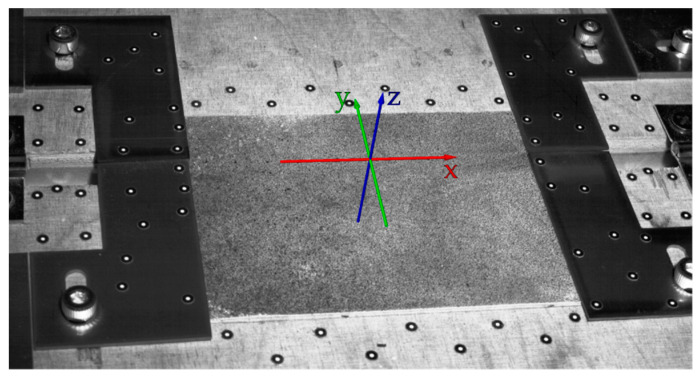
Position and orientation of the global coordinate system for the determination of the directional strains.

In order to analyze the temperature- and bending-induced local strain (positive deformation) or local compression (negative deformation), a global coordinate system was defined in the GOM ARAMIS Professional analysis software, whose origin is stationary with respect to the bending device (see Figure 7).

The *z*-axis (blue) was set perpendicular to the plane of the base plate (see Figure 7). The *y*-axis (green), also in plane with the surface of the base plate, is perpendicular to the edges of the bending and the base plate and is located midway between the mirrored pairs of positioning plates. The analysis software allows for measurement of the strains occurring on the visible surface of the film specimen in the global *x*- and *y*-directions, which follow the surface of the film tangentially, starting from the global coordinate systems’ position. An evaluation of the strain in the *x*-direction was not carried out. Of great interest, however, is the component of the deformation that occurs in the y-direction and provides the foundation for interpreting the film deformation in the course of the bending process. All the strains described are technical strains. Consequently, the basis for the strain calculation is always the original length corresponding to the reference image by default, taken at the beginning of each test. In the present study, the reference step was set to the time step before the initialization of the bending process in order to separate it from the preceding forming process. For the calculation of the strain ε, the following applies in general:

(1) $$\varepsilon =\frac{\Delta l}{l_{0}}.$$

In this context, the total length variation Δl is calculated by summing the individual length variations ($\Delta l_{i}$) between two image acquisition steps up to the current image acquisition step as follows:

(2) $$\Delta l=\Delta l_{1}+\Delta l_{2}+\ldots +\Delta l_{n}=\sum_{i=1}^{n}\Delta l_{i}.$$

Thus, the strain (ε) at the respective time of the *n*-image acquisition step is obtained, leading to:

(3) $$\varepsilon =\frac{\Delta l}{l_{0}}=\frac{\sum_{i=1}^{n}\Delta l_{i}}{l_{0}}.$$

Furthermore, it is possible to calculate the thermally induced elongation ($\varepsilon_{therm}$) due to the heating of the film material from the coefficient of linear thermal expansion ($\alpha_{therm}$) and the change in temperature (ΔT). For this purpose, the 2D temperature information captured by the IR camera was directly imported and mapped onto the measured 3D surface of the film in ARAMIS Professional. This mapping process links the temperature data to the homologous 3D coordinates so that each measured point can carry both temperature information from the corresponding IR image and strain information from the neighboring points. Based on the now given temperature distribution and its change over time on the film surface, the thermally induced strain ($\varepsilon_{therm}$) can be calculated as follows:

(4) $$\varepsilon_{therm}=\alpha_{therm}\cdot \Delta T.$$

The strain in the *y*-direction ($\varepsilon_{y}$) equals the sum of thermal and mechanical strain in the y-direction:

(5) $$\varepsilon_{y}=\varepsilon_{therm}+\varepsilon_{mech}.$$

Once $\varepsilon_{therm}$ and $\varepsilon_{mech}$ are calculated over the entire surface of the film specimen, the mechanical strain can be determined:

(6) $$\varepsilon_{mech}=\varepsilon_{y}-\varepsilon_{therm}.$$

## 3. Results and Discussion

In order to analyze the hot bending behavior of the film material and identify suitable parameter ranges, hot bending tests were carried out by varying certain process parameters. For this purpose, the effect of the heating duration ($t_{heat}$) as well as the applied heating power on the resulting forming ratio $\theta_{res}$ was investigated.

In the course of preliminary investigations, it became apparent that at a constant heating duration $t_{heat}∼$ 100 s, an applied heating power greater than 76.8 W led to damage of the film due to excessive heat flux. Therefore, for the following investigations, the heating duration was reduced in order to avoid damaging the material. Furthermore, the investigations of the PP-0.1 and TPU-0.3 configurations were carried out at shorter heating durations ($t_{heat}$ < 100 s) to assess a more efficient and less time-consuming parameter range. Since the initiation of the coupled measurement of the 3D DIC and IRT, the switching on and off of the power supply and the bending conversion were carried out manually, there were slight differences of $t_{heat}$ for some tests. For this reason, the actual heating durations were determined during the test evaluation with an initial temperature of the heating wire of 20°C for each test (see Table 2). Assuming that the electrical contact resistances and the resistances of the leads are small compared to the heating wire, a very small discrepancy between the heating power and the electrical power is expected.

For the PP-0.2 configuration, the maximum forming ratio was achieved at a heating duration of $t_{heat}$ = 102 s. However, the iterative reduction of heating energy with the aim of reducing the heating duration implied that a shorter heating duration and higher heating power led to similar values of the resulting forming ratio compared to a longer but less intense heating period (Table 2, Nos. 3–5). Therefore, the heating duration of the test configurations PP-0.1 and TPU-0.3 was reduced. For the configuration PP-0.1, the values of the resulting forming ratios lay in a close range with a maximum value of $\theta_{res}$ = 94.4% (Table 2, No. 8). In this context, the resulting forming ratios of film configuration TPU-0.3 did not differ significantly from the maximum forming ratio of $\theta_{res}$ = 66.3% (Table 2 No. 9–11). The fact that the resulting forming ratio of the TPU specimens is, in general, significantly lower than the corresponding values of the PP configurations is due to the lower stiffness of the TPU material. Therefore, the altering effect of gravity, pulling the top of the film downward, on the measured angle ($\alpha_{res}$), is greater for the TPU specimens compared to the PP specimens. In order to reduce this altering effect, the film area on top of the bending plate was shortened for all specimens. However, in order to enable a cross-material comparison, which was not the focus of the present study, a bending stiffness test has to be pursued to quantify and eliminate this altering effect [43].

### Identification of the Bending Area

**Figure 8:**
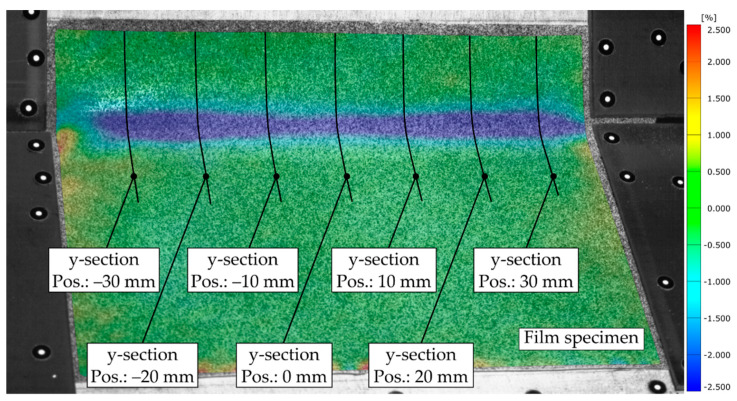
TPU-0.3 film at the time step after completion of the bending procedure showing the distribution of mechanical y-strain and the position of the y-sections enabling the identification of the bending area (test No. 9, t = 21.5 s).

The bending area is defined as the area where local mechanical deformation of the film specimens occurs as a result of the bending process. The determination of the bending area is of particular interest in order to determine the necessary area to be heated and to identify favorable temperature ranges within and at the boundaries of the bending area. In order to narrow down the region where mechanical deformation takes place, the total strain of the film must first be determined, as described in the Methods section. In the next step, within the analysis software, seven linear measuring sections were implemented, enabling the acquisition of the strains along the films’ surface (see Figure 8).

**Figure 9:**
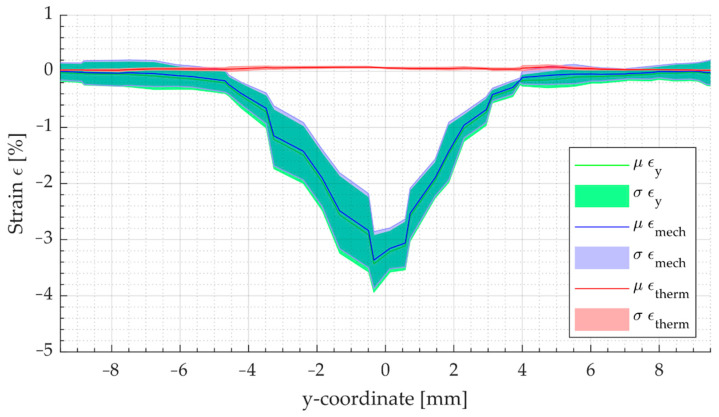
Plot of the mean values (μ) and the standard deviation (σ) of the total strain $\varepsilon_{y}$ (blue), thermal strain $\varepsilon_{therm}$ (red), and mechanical strain $\varepsilon_{mech}$ (green) for the measuring sections as a function of the y-coordinate of a TPU-0.3 specimen (test No. 9, t = 21.5 s).

Since the bending plate was lifted about the *x*-axis in the positive z-direction, the upper surface of the film was therefore the side where negative deformation (compression) occurred. Based on the mapped temperature distribution and the corresponding CTE for both materials, the thermal strain, and subsequently the mechanical strains, were determined. The curves of the y-strain $\varepsilon_{y}$ (blue), thermal strain $\varepsilon_{therm}$ (red), and mechanical y-strain $\varepsilon_{mech}$ (green) along the sections assigned to the measurement step are shown in Figure 9.

**Figure 10:**
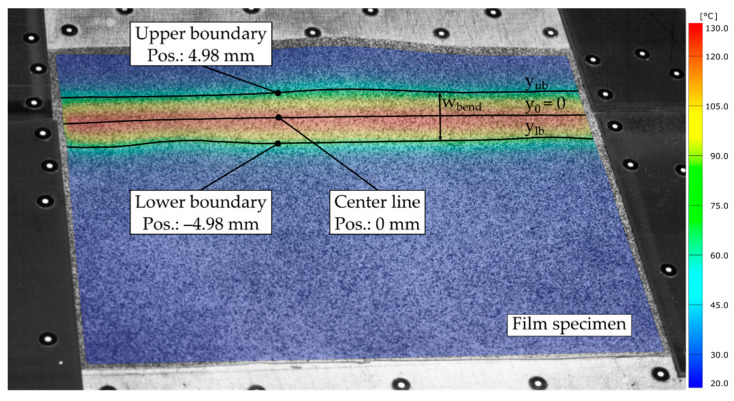
Temperature distribution on the upper surface of the film specimen and position of the measurement sections along the center as well as the upper and lower boundaries of the bending area one time step before initiating the bending procedure (test No. 9, t = 20.0 s).

In order to distinguish between the area with no strain and the area where bending-induced strain occurred, a threshold strain of −0.35% for the PP specimens and −0.25% for TPU were introduced, compensating for measurement uncertainties and noise. Subsequently, the rectangular bending area, running over the complete film length, was determined by arithmetic averaging of its y-component ($w_{bend}$) for the implemented sections of each specimen (see Figure 10).

To identify favorable process parameter ranges, the temperatures along the center line and the upper $y_{ub}=\frac{w_{bend}}{2}$ and lower $y_{lb}=-\frac{w_{bend}}{2}$ boundary of the bending area had to be determined. Since the bending plate and the base plate were symmetrically aligned with respect to the *x*-axis and the axis of rotation about which the hot bending occurs was parallel to the *x*-axis shifted in the z-direction, it was assumed that the bending area was also symmetrically located with respect to the *x*-axis. Thus, the measuring sections can be implemented parallel to the *x*-axis at the calculated positions along the boundaries and in the center of the bending area (see Figure 10).

Finally, the arithmetic mean value of the temperatures at the designated sections was captured one time step before initializing the bending procedure for the performed tests (see Table 3). Since shorter heating durations were aimed for, test Nos. 1 and 2 were not considered.

For the majority of tests, the upper boundary temperature $\vartheta_{ub}$ had a lower value than the lower boundary temperature $\vartheta_{lb}$ (see Table 3). Investigations regarding the arithmetic temperature means over the length of the bending area in the x-direction showed that, with a few exceptions, the maximum temperature did not run along the *x*-axis, but slightly shifted in the negative y-direction. In this context, a maximum offset of the maximum temperature of $\Delta y_{offset,max}$ = −0.7 mm occurs. This is possibly due to an offset of the heating wire during the execution of the experiment that needs to be considered in the conceptualization of future manufacturing processes.

The primary objective of this study was to identify the parameter combination giving the largest possible resulting forming ratios. The second object of investigation was to identify the process parameter ranges that lead to similar forming ratios through a more efficient heating process, taking time and energy consumption into account. The selection of suitable values for the process parameters initially depends on the specific objective of the manufacturing process. Therefore, it has to be clarified individually whether energy saving or the realization of the highest possible forming ratio has a higher priority. The ranges of the process parameter investigated were applied within the experimental tests and the corresponding resulting forming ratios of each film configuration are listed in Table 4. If the process targets a maximum forming ratio, the upper limit of the parameter range should be taken into account. Otherwise, the lower limit should be applied as a recommended reference for reducing the energy consumption. For the boundary temperature ranges given, the lower temperature value was considered for each test carried out (see Table 3).

**Figure 11:**
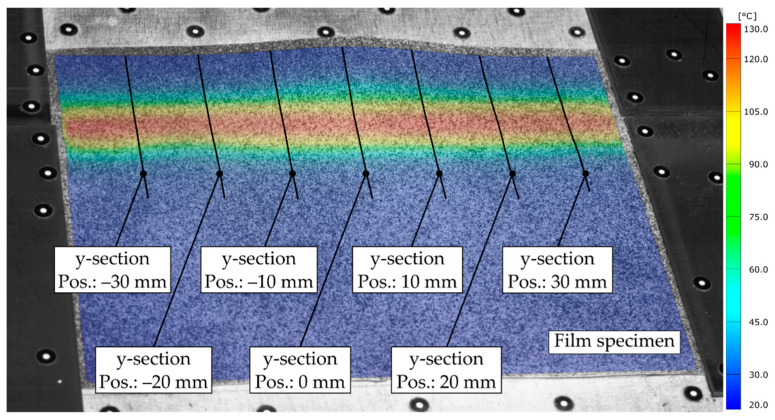
TPU-0.3-specimen with mapped temperature field and measurement sections (test No. 9, t = 20.0 s).

To assess the effect of thermal expansion of the film material within a serial process, the thermally induced elongation Δl was determined with respect to the reference image at the beginning of the test, using the aforementioned measurement sections (see Figure 11).

The figure shows the TPU-0.3 specimen of test No. 9 with the mapped temperature field immediately before the bending plate is lifted during the bending process. At this point, the measured thermal strain along the sections reaches its maximum value. Since no mechanical deformation due to the bending process has taken place up to this point, the measured length variations are due to thermal and gravimetric effects. In the context of the continuous fabrication of novel cellular structures, it is important to note that hot bending must occur at multiple locations across the width of a continuously moving film sheet. Consequently, there is a width variation ($\Delta w_{film}$) across the direction of motion of the film, resulting from its thermal expansion and number of bending areas. In order to obtain a uniform width of the bending area over its complete length, the length variation ($\Delta l_{avg}$) measured along the sections was averaged. Considering $\Delta l_{avg}$ determined in test No. 9 and a feasible number of 20 bending areas along the width of the film, the total width variation of the film results as follows:

(7) $$\Delta w_{film}=20\cdot \Delta l_{avg}=20\cdot 0.33\;\mathrm{mm}=6.60\;\mathrm{mm}.$$

The displacement of the individual hot bending areas due to $\Delta w_{film}$ must be considered for the realization of precise forming in a continuous process.

## 4. Conclusions

In this work, coupled IRT and DIC analysis was applied to investigate the effect of the process parameters of heating power as well as heating duration on the forming ratio of thermoplastic films in bending tests. The combined method of simultaneous deformation and temperature recording during the forming process enables a wide range of possibilities for process analysis. Thus, the actual mechanically stressed area of the film as a result of the bending process could be identified, allowing the determination of the necessary local forming temperature for the desired forming ratios. The subject of further investigations should be to reduce the thermal impact on the film material while maintaining the highest possible forming ratio. The favorable process parameters determined (see Table 4) and the side effects such as the width variation of the film due to heating will serve as the starting point for future process analyses on a larger scale.

## Figures and Tables

**Table 1:** Material and geometric parameters of the investigated film materials.

Property	Unit	Thermoplastic Polyurethane Film	Polypropylene-Based Film
$\mathrm{Degradation}\;\mathrm{temperature}\;\vartheta_{D}$	°C	324.1 ^1^	444.5 ^1^
$\mathrm{Melting}\;\mathrm{range}\;\vartheta_{M}$	°C	162.8–216.8 ^2^	98.9–166.6 ^2^
Density ρ	g/cm^3^	1.08	0.89
Thickness *t*	mm	0.3	0.1, 0.2

^1^ Determined in thermogravimetric analysis according to DIN EN ISO 11358-1.

^2^ Determined in differential scanning calorimetry analysis according to DIN EN ISO 11357-1.

**Table 2:** Resulting forming ratio for different film configurations and heating parameters.

No.	Film Configuration	Heating Power	Heating Duration	$\mathrm{Resulting}\;\mathrm{Forming}\;\mathrm{Ratio}\;\theta_{res}$	Standard Deviation
		[W]	[s]	[%]	[%]
1	PP-0.2	48.5	100.0	86.9	±3.3
2	PP-0.2	53.0	102.0	93.4	
3	PP-0.2	76.8	14.5	83.7	
4	PP-0.2	82.5	10.0	86.6	
5	PP-0.2	89.7	16.0	90.1	
6	PP-0.1	64.9	11.0	88.9	±4.2
7	PP-0.1	76.8	9.0	86.1	
8	PP-0.1	76.8	11.0	94.4	
9	TPU-0.3	82.5	20.0	63.9	±1.2
10	TPU-0.3	89.0	25.0	63.6	
11	TPU-0.3	103.6	24.5	66.3	

**Table 3:** Average temperature at the boundaries and at the center line of the hot bending area for a selection of tests.

No.	Film Config.	Heating Power	Heating Duration	Boundaries Y-Coordinate	Temp. Upper Boundary	Temp. Lower Boundary	Temp. Center Line
		[W]	[s]	[mm]	[°C]	[°C]	[°C]
3	PP-0.2	76.8	14.5	±3.15	85.2	86.5	103.5
4	PP-0.2	82.5	10.0	±3.30	74.0	77.6	94.4
5	PP-0.2	89.7	16.0	±3.75	85.7	90.8	111.5
6	PP-0.1	64.9	11.0	±3.49	78.1	79.2	101.0
7	PP-0.1	76.8	9.0	±3.05	82.4	85.8	103.4
8	PP-0.1	76.8	11.0	±2.78	91.3	98.0	110.8
9	TPU-0.3	82.5	20.0	±4.98	59.9	74.5	123.2
10	TPU-0.3	89.0	25.0	±5.44	65.5	65.5	154.5
11	TPU-0.3	103.6	24.5	±5.65	65.7	72.7	153.2

**Table 4:** Value ranges of process parameters and the corresponding boundary temperatures and forming ratios.

Process Parameter	Unit	PP-0.2	PP-0.1	TPU-0.3
Heating power	W	76.8 … 89.7	64.9 … 76.8	82.5 … 103.6
Heating duration	s	10.0 … 16.0	9.0 … 11.0	20.0 … 25.0
Heating energy	kJ	0.83 … 1.44	0.69 … 0.84	1.65 … 2.54
Resulting forming ratio	%	86.6 … 90.1	86.1 … 94.4	63.6 … 66.3
Minimum boundary temperature	°C	74.0 … 85.2	78.1 … 91.3	59.9 … 65.7

## Data Availability

The data presented in the current study are available on request from the corresponding author.
